# Adaptation of cucumber seedlings to low temperature stress by reducing nitrate to ammonium during it’s transportation

**DOI:** 10.1186/s12870-021-02918-6

**Published:** 2021-04-19

**Authors:** Yumei Liu, Longqiang Bai, Mintao Sun, Jun Wang, Shuzhen Li, Li Miao, Yan Yan, Chaoxing He, Xianchang Yu, Yansu Li

**Affiliations:** 1 The Institute of Vegetables and Flowers, Chinese Academy of Agricultural Sciences, Beijing, 100081 China; 2 College of Agricultural and Biological Engineering, Heze University, Heze, 274000 Shandong China; 3 College of Horticulture, Shanxi Agricultural University, Taigu, 030801 Shanxi China; 4 College of Life Science, Gannan Normal University, Ganzhou, 341000 Jiangxi China

**Keywords:** Cucumber, Low temperature, Nitrate, Ammonium, Transportation

## Abstract

**Background:**

Low temperature severely depresses the uptake, translocation from the root to the shoot, and metabolism of nitrate and ammonium in thermophilic plants such as cucumber (*Cucumis sativus*). Plant growth is inhibited accordingly. However, the availability of information on the effects of low temperature on nitrogen transport remains limited.

**Results:**

Using non-invasive micro-test technology, the net nitrate (NO_3_^−^) and ammonium (NH_4_^+^) fluxes in the root hair zone and vascular bundles of the primary root, stem, petiole, midrib, lateral vein, and shoot tip of cucumber seedlings under normal temperature (NT; 26 °C) and low temperature (LT; 8 °C) treatment were analyzed. Under LT treatment, the net NO_3_^−^ flux rate in the root hair zone and vascular bundles of cucumber seedlings decreased, whereas the net NH_4_^+^ flux rate in vascular bundles of the midrib, lateral vein, and shoot tip increased. Accordingly, the relative expression of *CsNRT1.4a* in the petiole and midrib was down-regulated, whereas the expression of *CsAMT1.2a*–*1.2c* in the midrib was up-regulated. The results of ^15^N isotope tracing showed that NO_3_^−^-N and NH_4_^+^-N uptake of the seedlings under LT treatment decreased significantly compared with that under NT treatment, and the concentration and proportion of both NO_3_^−^-N and NH_4_^+^-N distributed in the shoot decreased. Under LT treatment, the actual nitrate reductase activity (NRA_act_) in the root did not change significantly, whereas NRA_act_ in the stem and petiole increased by 113.2 and 96.2%, respectively.

**Conclusions:**

The higher net NH_4_^+^ flux rate in leaves and young tissues may reflect the higher NRA_act_ in the stem and petiole, which may result in a higher proportion of NO_3_^−^ being reduced to NH_4_^+^ during the upward transportation of NO_3_^−^. The results contribute to an improved understanding of the mechanism of changes in nitrate transportation in plants in response to low-temperature stress.

**Supplementary Information:**

The online version contains supplementary material available at 10.1186/s12870-021-02918-6.

## Background

Cucumber (*Cucumis sativus* L.) is an important vegetable crop worldwide and a model plant system for studying sex determination and vascular biology [1]. It is native to the tropics and is sensitive to low temperature [2]. Cucumber is widely cultivated in greenhouses in northern China during the winter and spring seasons. Low temperature is a crucial environmental factor that limits the development and productivity of cucumber crops [3].

Nitrogen (N) is the mineral nutrient required in the highest amount by plants [4]. It is crucial for the biosynthesis of amino acids, proteins, and nucleic acids [5]. Nitrogen contributes approximately 2% of plant dry matter and exerts the greatest nutrient influence (up to 50%) on the growth and yield of plants [6, 7]. The absorption and utilization of N by plants under normal temperatures have been clarified. Plant roots absorb N primarily as nitrate (NO_3_^−^) and ammonium (NH_4_^+^), especially as NO_3_^−^ for terrestrial plants [8]. The NO_3_^−^ absorbed by plants is first reduced to NH_4_^+^ before it can be metabolized. This reduction is catalyzed by nitrate reductase (NR) and nitrite reductase (NiR) [9]. Of these enzymes, NR is considered to be the rate-limiting step in N assimilation [10]. Activity of NR and NiR can be detected in many plant organs (e.g., the root, stem, cotyledon, inflorescence stalk, flower, petiole, and leaf) [11–14].

The absorption and transportation of NO_3_^−^ and NH_4_^+^ in plants is mediated by nitrate transporters (NRTs) and ammonium transporters (AMTs), respectively [15]. Four families of nitrate-transporting proteins have been identified to date: nitrate transporter 1 family (NRT1), nitrate transporter 2 family (NRT2), chloride channel family (CLC), and slow anion channel-associated homologs (SLAC1/SLAH) [16]. The ammonium transporter gene family of vascular plants consists of two clades, comprising AMTs and methylammonium permeases (MEPs) [17]. The regulation of NO_3_^−^ uptake and transport is often highly correlated with changes in expression of relevant transporter genes [18, 19].

The uptake of inorganic N forms is favored by warm temperatures, especially NO_3_^−^ uptake [20]. In many crop species, particularly those originating from tropical and subtropical regions, low temperature restricts the uptake capacity of the root and distribution of NO_3_^−^ and NH_4_^+^ in the shoot and, consequently, plant growth and metabolism are inhibited [21–23]. Furthermore, reduction in temperature decreases N translocation more strongly than uptake and, as a result, the N concentration in the root increases [24]. However, previous studies mainly focused on the changes in physiological characteristics under low temperature. Hence the molecular mechanism of these phenomena remains unclear. Limited information is available on the adaptation of NO_3_^−^ and NH_4_^+^ transport in vascular bundles under low-temperature stress.

The use of stable N isotopes has typically been the most important method in the study of N absorption and transportation, but only enables monitoring of N absorption and distribution within a certain period. It is difficult to monitor the dynamic transport of N. No effective technology to study N transport under low temperature has previously existed. In recent years, non-invasive micro-test technology (NMT) has provided a novel means to detect ion velocity in living plant tissues [25]. The development and application of NO_3_^−^ and NH_4_^+^ sensors for NMT provide convenience for intuitive detection of net NO_3_^−^ and NH_4_^+^ flow rates [26, 27]. The purpose of the present study was to study the effects of low temperature on the absorption and transportation of NO_3_^−^ and NH_4_^+^, treating the plant as a whole using NMT technology, in combination with ^15^N isotope tracing and quantitative reverse transcription–PCR (qRT-PCR) technology.

We observed that low temperature reduced the net NO_3_^−^ flux rate in the root hair zone and vascular bundles of cucumber seedlings, whereas the net NH_4_^+^ flux rate was enhanced in vascular bundles of the midrib, lateral vein, and shoot tip. To further understand the regulation of N transportation by low temperature, the uptake and distribution of ^15^N-NO_3_^−^ and ^15^N-NH_4_^+^, NR and NiR activities and gene expression, and relative expression of *CsNRT* and *CsAMT* were measured under normal temperature (NT) and low temperature (LT) treatments. The results will aid in understanding the adaptability of inorganic N transport in thermophilic plants to low-temperature stress.

## Results

### Net NO_3_^−^ and NH_4_^+^ flux rates

**Fig. 1:**
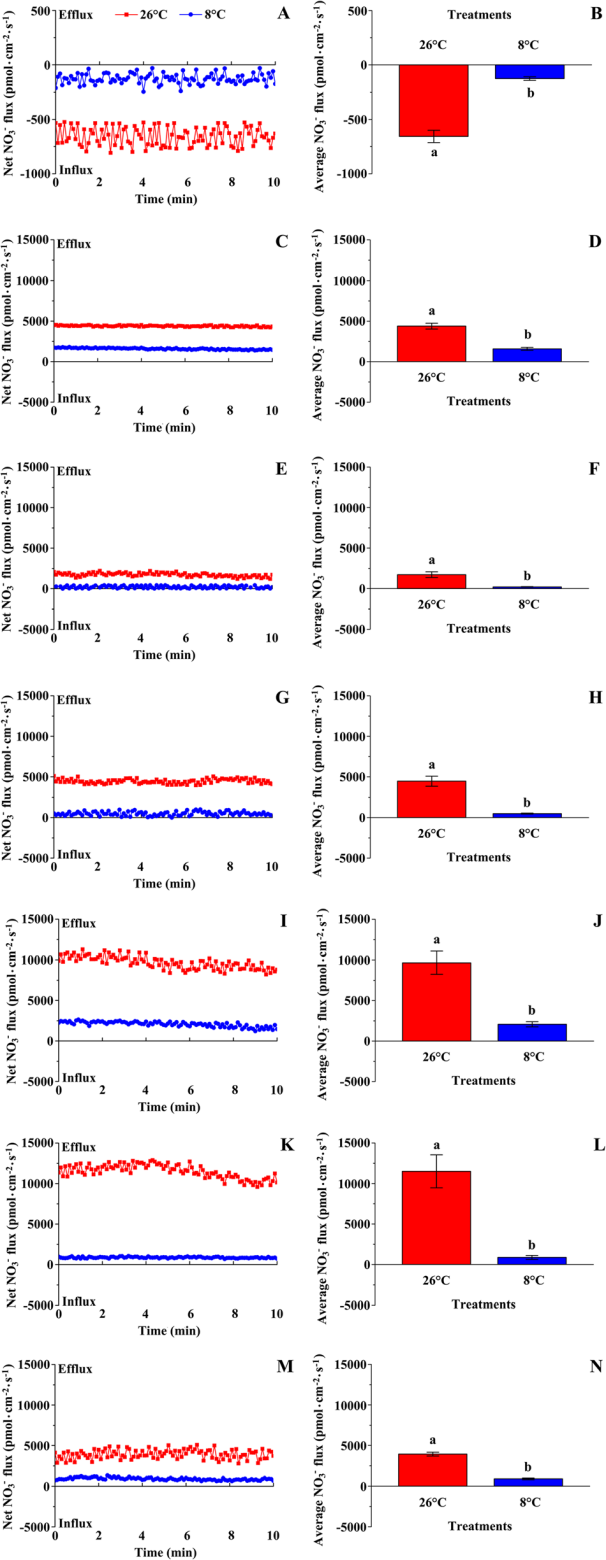
Effects of low temperature on net NO_3_^−^ fluxes of cucumber seedlings. The net NO_3_^−^ flux rate in the root hair zone (A), and vascular bundles of the primary root (C), stem (E), petiole (G), midrib (I), lateral vein (K), and shoot tip (M). The average NO_3_^−^ flux rate in the root hair zone (B), and vascular bundles of the primary root (D), stem (F), petiole (H), midrib (J), lateral vein (L), and shoot tip (N). Steady-state fluxes of NO_3_^−^ and NH_4_^+^ were examined by continuous flux recording for 10 min (*n* = 5). Different lower-case letters indicate a significant difference (*P <* 0.05)

First, we observed that LT (8 °C) treatment significantly depressed the net NO_3_^−^ flux rate in the root hair zone and in the vascular bundles of other organs of cucumber seedlings. Compared with NT (26 °C) treatment, the net NO_3_^−^ influx rate in the root hair zone and the net NO_3_^−^ efflux rate in the vascular bundles of the primary root, stem, petiole, midrib, lateral vein, and shoot tip under LT treatment decreased significantly (Fig. 1), indicating that the uptake and upward transport of NO_3_^−^ was significantly inhibited by low temperature.

**Fig. 2:**
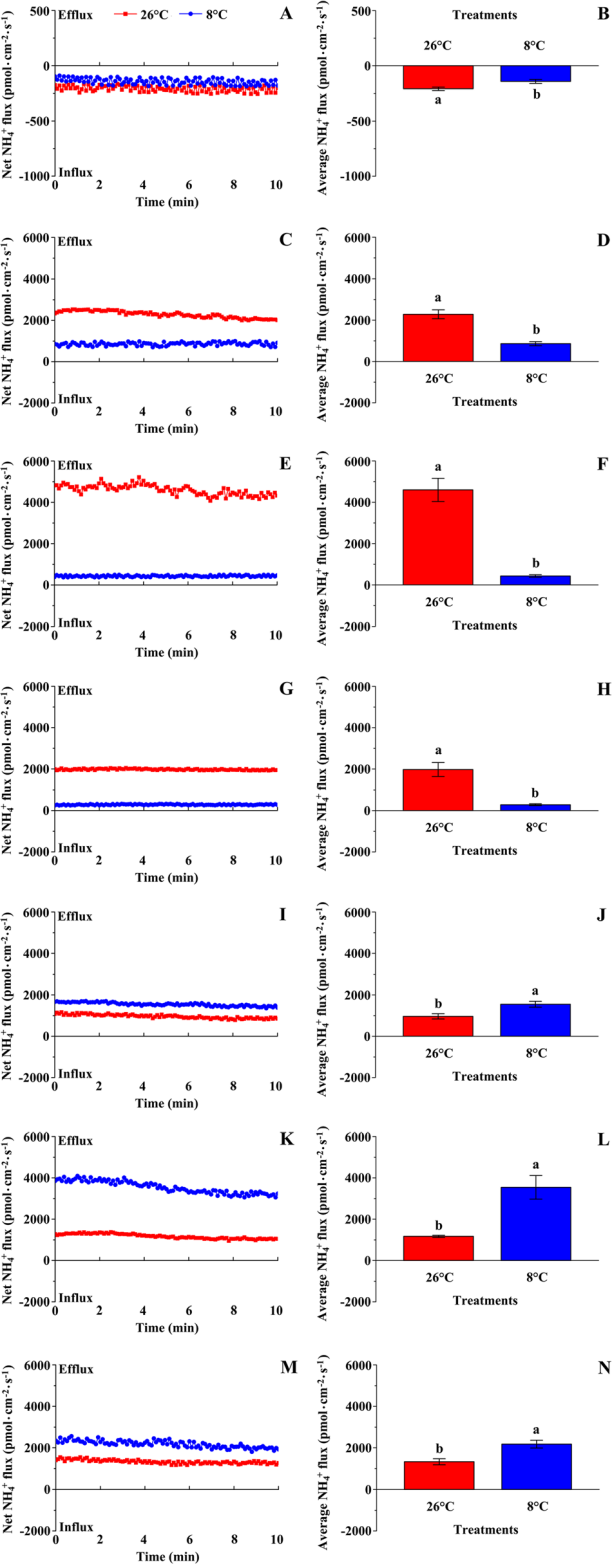
Effects of low temperature on net NH_4_^+^ fluxes of cucumber seedlings. The net NH_4_^+^ flux rate in the root hair zone (A), and vascular bundles of the primary root (C), stem (E), petiole (G), midrib (I), lateral vein (K), and shoot tip (M). The average NH_4_^+^ flux rate in the root hair zone (B), and vascular bundles of the primary root (D), stem (F), petiole (H), midrib (J), lateral vein (L), and shoot tip (N). Steady-state fluxes of NO_3_^−^ and NH_4_^+^ were examined by continuous flux recording for 10 min (*n* = 5). Different lower-case letters indicate a significant difference (*P <* 0.05)

Compared with the net NO_3_^−^ flux rate, the change in net NH_4_^+^ flux rate under LT treatment was different. The net NH_4_^+^ flux rate in the root hair zone and vascular bundles of the primary root, stem, and petiole of cucumber seedlings under LT treatment decreased significantly compared with those under NT treatment. In contrast, the net NH_4_^+^ flux rate in the vascular bundles of the midrib, lateral vein, and shoot tip increased significantly (Fig. 2).

Compared with the net NO_3_^−^ flux rate, the net NH_4_^+^ flux rate at the detection sites was markedly lower under NT treatment, but significantly higher in the lateral vein and shoot tip under LT treatment. These results indicated that the inhibition of net NO_3_^−^ flux rate at low temperature was more severe than the effect on net NH_4_^+^ flux rate.

### Nitrogen uptake per plant, N concentration, and N distribution in different organs

The effects of low temperature on the uptake and distribution of NO_3_^−^-N and NH_4_^+^-N in cucumber seedlings were further explored using an isotope tracer method. Compared with NT treatment, NO_3_^−^-N, NH_4_^+^-N, and total N uptake per plant under LT treatment decreased significantly (Table 1). These effects were consistent with the change in net NO_3_^−^ and NH_4_^+^ flux rates in the root hair zone under LT treatment (Figs. 1 and 2). Under LT treatment, the ratio of NO_3_^−^-N to total N decreased significantly, whereas the ratio of NH_4_^+^-N to total N increased significantly, compared with those under NT treatment. This result indicated that, compared with NH_4_^+^-N uptake, low temperature inhibited NO_3_^−^-N uptake more severely.

**Table 1:** NO_3_^−^-N, NH_4_^+^-N, and total N uptake per cucumber seedling exposed to 26 °C or 8 °C for 5 h

Treatment	NO_3_^−^-N uptake (μmol per plant)	NH_4_^+^-N uptake (μmol per plant)	Total N uptake (μmol per plant)	NO_3_^−^-N/total N (%)	NH_4_^+^-N/total N (%)
NT(26 °C)	229.60 ± 12.13 a	92.13 ± 6.60 a	321.73 ± 18.73 a	71.37 ± 3.78 a	28.63 ± 1.86 b
LT(8 °C)	50.33 ± 3.00 b	37.93 ± 2.33 b	88.27 ± 5.33 b	57.02 ± 3.36 b	42.98 ± 2.53 a

*NT* Normal temperature, *LT* Low temperature. Total N refers to NO_3_^−^-N plus NH_4_^+^-N. Values are the mean ± SE (*n* = 3). Different lower-case letters within a column indicate a significant difference (*P <* 0.05)

**Fig. 3:**
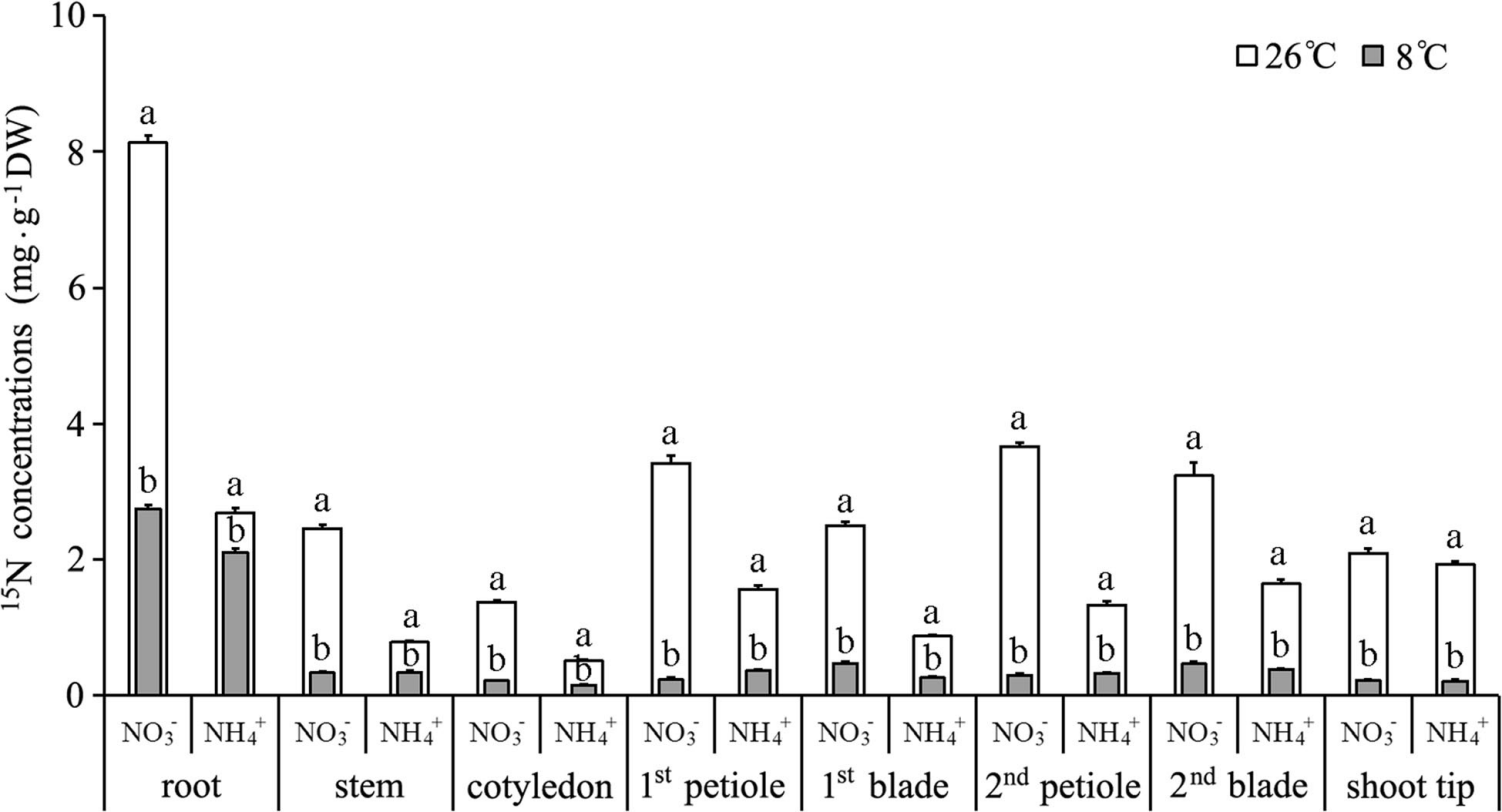
Effects of low temperature on NO_3_^−^-N and NH_4_^+^-N concentrations in cucumber seedlings. Error bars represent the standard error of the mean (*n* = 3). Different lower-case letters indicate a significant difference (*P <* 0.05)

Under NT treatment, the NO_3_^−^-N concentrations at the detection sites of cucumber seedlings were significantly higher than NH_4_^+^-N concentrations (Fig. 3), indicating that NO_3_^−^-N is the predominant form of N used by cucumber seedlings. Compared with those under NT treatment, the NO_3_^−^-N and NH_4_^+^-N concentrations in different organs under LT treatment decreased significantly. Low temperature led to a smaller decrease in NH_4_^+^-N concentrations compared with that for NO_3_^−^-N concentrations. This result was consistent with Figs. 1 and 2.

**Fig. 4:**
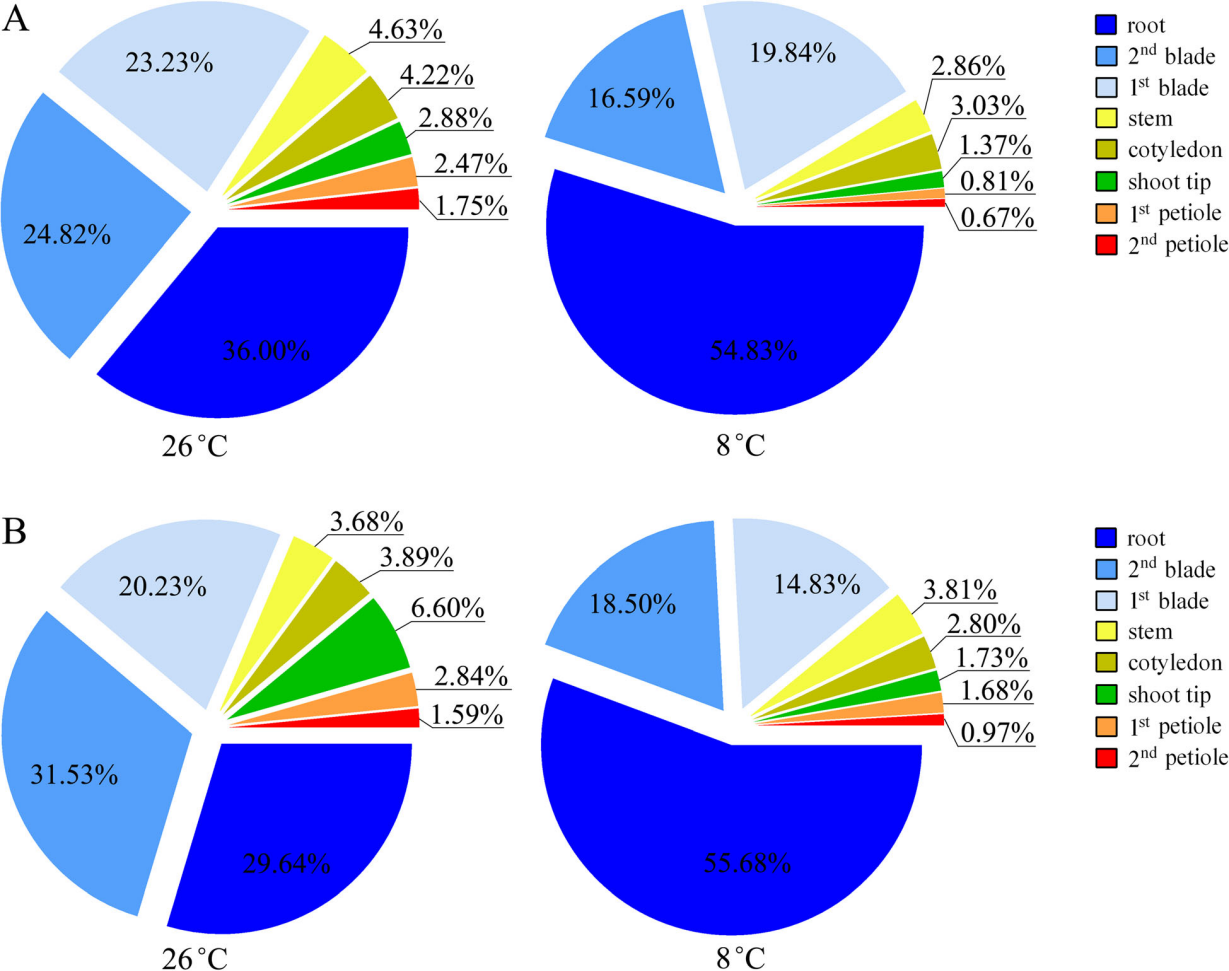
Effects of low temperature on NO_3_^−^-N (A) and NH_4_^+^-N (B) distribution in cucumber seedlings. The values are means (*n* = 3)

Exposure of cucumber seedlings to low temperature resulted in a significant increase in not only NO_3_^−^-N but also NH_4_^+^-N distribution proportion in the root (Fig. 4). Thus, LT treatment significantly reduced the distribution proportion of NO_3_^−^-N and NH_4_^+^-N in the shoot. This finding indicated that low temperature inhibited the transportation of NO_3_^−^ and NH_4_^+^ from the root to the shoot, and resulted in N accumulation in the root. Under LT treatment, the distribution proportion of NO_3_^−^-N and NH_4_^+^-N in all aerial organs decreased, except for the NH_4_^+^-N distribution proportion in the stem.

### Expression of *CsNRT* and *CsAMT* genes in the petiole and midrib

**Fig. 5:**
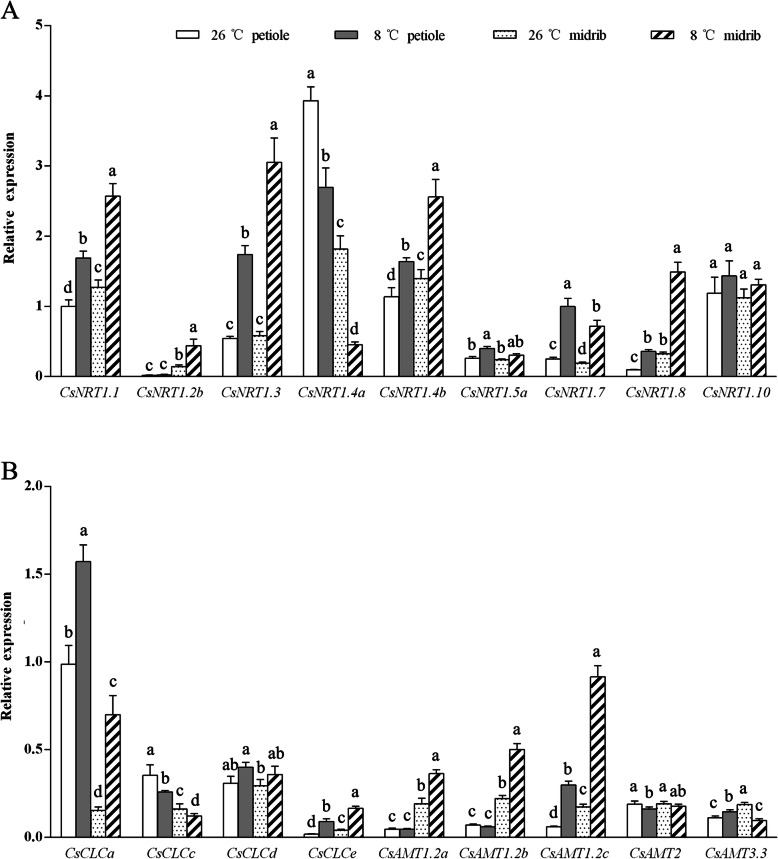
Effect of low temperature on expression levels of NRT and AMT family genes in cucumber seedlings. (A) Expression profiles of *CsNRT* genes. (B) Expression profiles of *CsCLC* and *CsAMT* genes. Error bars represent the standard error of the mean (*n* = 3). Different lower-case letters indicate a significant difference (*P <* 0.05)

To investigate the response of *CsNRT* and *CsAMT* genes in cucumber seedlings to low temperature, the relative expression of 34 genes was quantified. Exposure to low temperature decreased expression of *CsNRT1.4a* in the petiole and midrib significantly, whereas the expression levels of *CsAMT1.2a*, *CsAMT1.2b*, and *CsAMT1.2c* were significantly enhanced in the midrib (Fig. 5). Thus, the expression of these genes may be strongly associated with NO_3_^−^ and NH_4_^+^ transport in the petiole and midrib. Compared with those under NT treatment, the expression levels of *CsNRT1.1*, *CsNRT1.3*, *CsNRT1.7*, *CsNRT1.8*, *CsCLCa*, and *CsCLCe* in the petiole and midrib, and that of *CsNRT1.2b* in the midrib were up-regulated. Therefore, these genes were not indicated to play crucial roles in nitrate transport in the petiole and midrib.

The relative expression of *CsNRT1.2a*, *CsNRT1.5a*, *CsNRT1.10*, *CsCLCc*, *CsCLCd*, *CsAMT2*, and *CsAMT3.3* in the petiole and midrib, and those of *CsNRT1.2b*, *CsNRT1.4a*, *CsNRT1.4b*, *CsCLCa*, *CsCLCb*, *CsAMT1.2a*, and *CsAMT1.2b* in the petiole were not significantly affected by LT treatment. The relative expression levels of *CsNRT1.2c*, *CsNRT1.5b*, *CsNRT1.5c*, *CsNRT1.9*, *CsSLAH1–4*, *CsCLCf*, *CsCLCg*, *CsAMT1.1a*, and *CsAMT1.1b* in the petiole and midrib under the NT and LT treatments were substantially lower than those of the genes shown in Fig. 5. Therefore, data on their relative expression levels are not presented.

### Activities of NR and NiR, and expression of *CsNR* and *CsNiR* genes

**Fig. 6:**
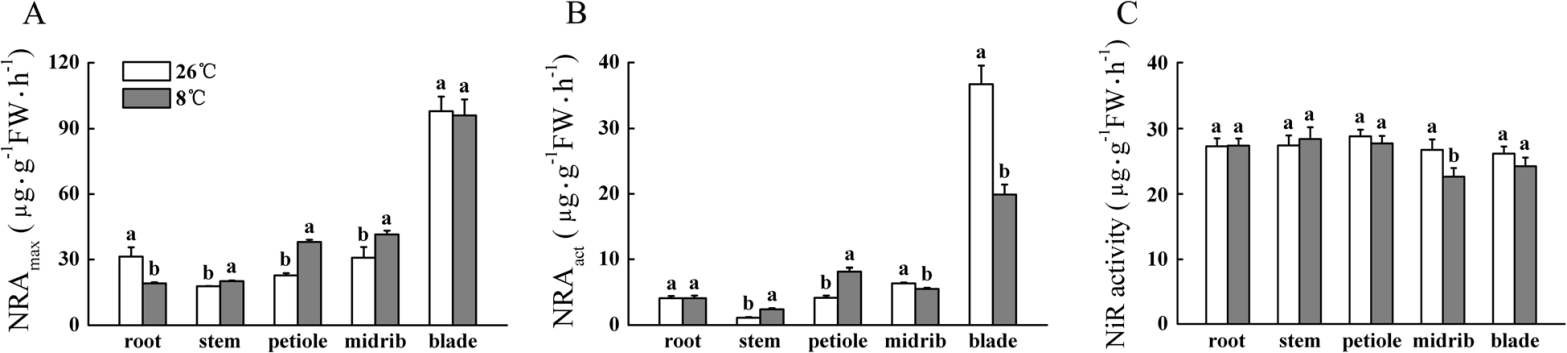
Effect of low temperature on nitrate reductase (NR) and nitrite reductase (NiR) activities of cucumber seedlings. (A) NRA_max_, the maximum activity of nitrate reductase; (B) NRA_act_, the actual activity of nitrate reductase; (C) NiR activity, the activity of nitrite reductase. Error bars represent the standard error of the mean (*n* = 3). Different lower-case letters indicate a significant difference (*P <* 0.05)

The enzymes NR and NiR catalyze the nitrate-to-nitrite and nitrite-to-ammonium reduction processes, respectively, in plants [10, 28]. The NRA_max_ reflects the maximum amount of enzyme protein indirectly and NRA_act_ indicates actual NR activity in situ [29]. After LT treatment for 5 h, NRA_max_ in the root decreased significantly, whereas that in the stem, petiole, and midrib increased significantly, compared with that under NT treatment (Fig. 6A). No significant difference in NRA_max_ in the blade was observed between the two treatments. Compared with that under NT treatment, NRA_act_ in the stem and petiole increased significantly, whereas that in the midrib and blade decreased significantly under LT treatment (Fig. 6B). No significant difference in NRA_act_ in the root was observed between the NT and LT treatments. The NiR activity in the root, stem, petiole, and blade was not significantly decreased by LT treatment, except for the midrib (Fig. 6C).

**Fig. 7:**
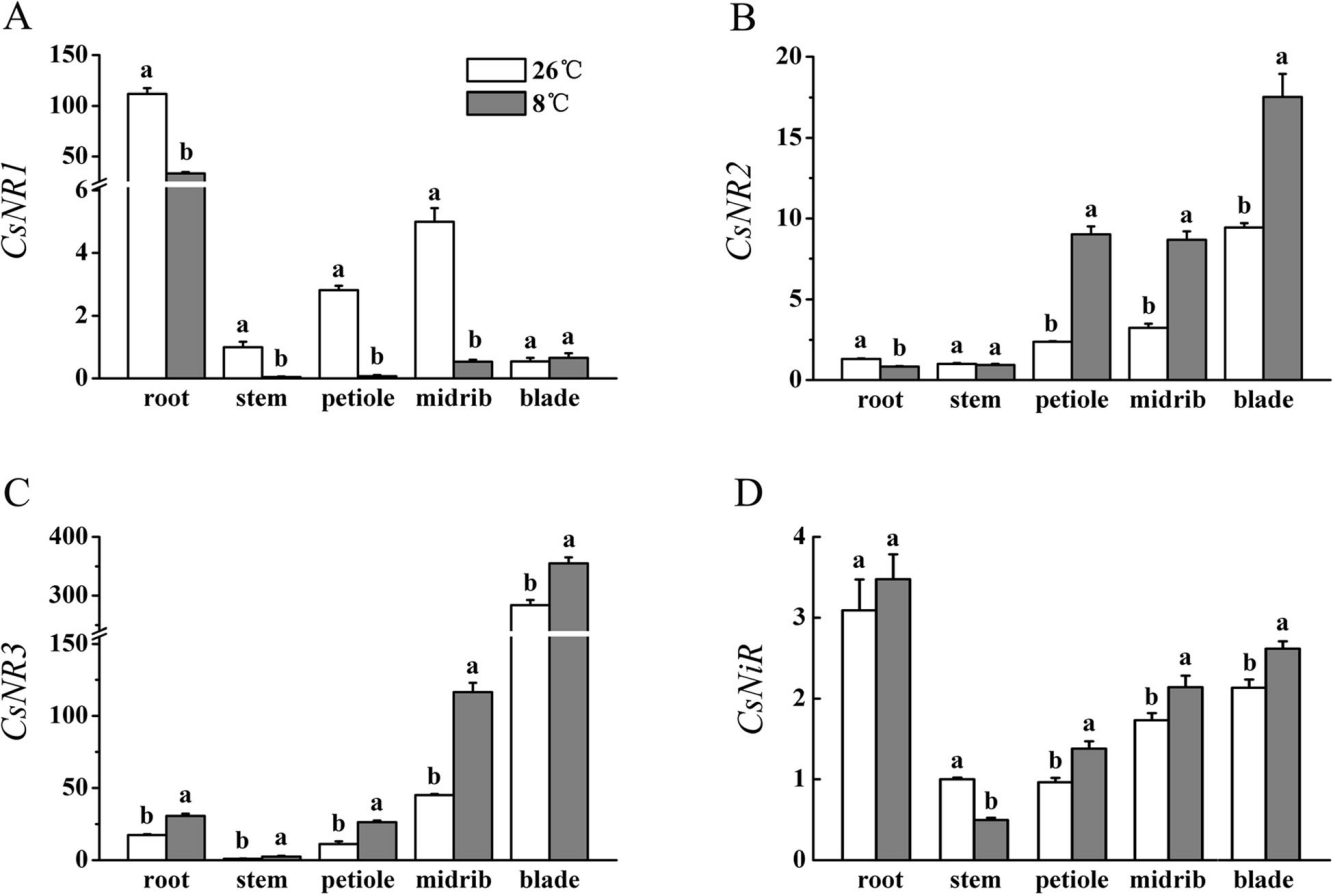
Effect of low temperature on relative expression of *CsNR* genes and *CsNiR* of cucumber seedlings. Error bars represent the standard error of the mean (*n* = 3). Different lower-case letters indicate a significant difference (*P <* 0.05)

The *C. sativus* genome contains three NR family genes (*CsNR1*, *CsNR2*, and *CsNR3*) according to Reda et al. [30]. Compared with that under NT treatment, expression of *CsNR1* in the root, stem, petiole, and midrib was down-regulated under LT treatment, whereas no significant difference in relative expression of *CsNR1* in the blade was observed between the NT and LT treatments (Fig. 7A). Under LT treatment, expression of *CsNR2* in the leaf (including the petiole, midrib, and blade) was up-regulated, whereas expression of *CsNR2* in the root decreased, compared with that under NT treatment (Fig. 7B). No significant difference in *CsNR2* expression in the stem was observed between the LT and NT treatments. Compared with that under NT treatment, expression of *CsNR3* in the root, stem, petiole, and midrib under LT treatment was up-regulated (Fig. 7C). The relative expression of *CsNR1* in the root was substantially higher than that in other organs under the NT and LT treatments (Fig. 7A). The relative expression of *CsNR2* in the leaf was higher than that in the root and stem (Fig. 7B). Similar to *CsNR2*, a high expression level for *CsNR3* was observed in the midrib, and especially in the blade, under the NT and LT treatments. The results presented in Figs. 6A and 7A, B, C suggested that *CsNR1* may be the dominant NR gene expressed in the root, and that *CsNR3* may be the dominant NR gene expressed in the leaf. *CsNR2* and *CsNR3* may play a leading role together in the stem and petiole.

Compared with *CsNR* genes, the differences in relative expression levels of *CsNiR* among different organs were small (Fig. 7D). The LT treatment enhanced the expression of *CsNiR* in the petiole, midrib, and blade to a certain extent. The highest expression level of *CsNiR* was observed in the root in both NT and LT treatments, but its expression was not affected significantly by low temperature.

## Discussion

### Low temperature inhibited NO_3_^−^ and NH_4_^+^ uptake and upward transportation, but increased net NH_4_^+^ efflux rate in the midrib, lateral vein, and shoot tip of cucumber seedlings

Hessini et al. [31] reported that cucumber preferentially absorbs NO_3_^−^-N rather than NH_4_^+^-N as the compound N source under normal environmental conditions. The present results confirmed that under NT treatment, NO_3_^−^-N was the main N form used by cucumber seedlings. Previous studies have shown that the effects of low temperature on the uptake of NO_3_^−^-N and NH_4_^+^-N are different. Root temperature affects the kinetic parameters of NO_3_^−^ uptake more than those of NH_4_^+^ uptake in *Ceratonia siliqua* [32]. For barley, Q10 temperature coefficients for NO_3_^−^ are higher than those for NH_4_^+^ [33]. Under low temperature the NO_3_^−^ uptake in *Secale cereale* and *Brassica napus* is reduced [34]*.* The current results showed that, compared with cucumber seedlings grown under 26 °C, the NO_3_^−^-N and NH_4_^+^-N absorbed by cucumber seedlings under low temperature (8 °C) decreased significantly, especially NO_3_^−^ (Table 1), indicating that the inhibition of low temperature on NO_3_^−^ uptake was greater than that of NH_4_^+^ uptake. This may be because uptake of NO_3_^−^ is energy dependent [35]. The energy requirements for absorption and assimilation of NO_3_^−^ are several-fold higher than those of NH_4_^+^ [36]. With the occurrence of low-temperature stress, the energy absorbed and utilized by leaves decreased significantly [37]. Thus, under LT treatment, the uptake of NO_3_^−^ by roots would be severely inhibited as a result of energy limitation.

The transport of NO_3_^−^ is induced by NO_3_^−^ itself and promoted by photosynthesis [38]. Under low temperature the xylem sap transport in cucumber is reduced severely [39]. Laine et al. [34] reported that low temperature decreases xylem N translocation and results in N accumulation in the roots of *Secale cereale* and *Brassica napus*. The present results confirmed that low temperature not only inhibited uptake of NO_3_^−^-N and NH_4_^+^-N, but also inhibited their upward transportation. The degree of inhibition of NO_3_^−^-N upward transportation was almost identical to that of NH_4_^+^-N under low temperature. Recently, Anwar et al. [23] reported that low temperature reduces N content in roots of cucumber seedlings, but does not significantly reduce N content in the shoot. This conflict may be due to the different detection methods used. The total N contents were detected by Anwar et al. [23], whereas the ^15^N concentrations were detected in the present experiment.

The net NO_3_^−^ and NH_4_^+^ flux rates detected by NMT showed that, compared with NT treatment, the change in net NO_3_^−^/NH_4_^+^ flux rate in the stem and petiole under LT treatment differed from that in the leaf vein and shoot tip. In contrast to the change in net NO_3_^−^ flux rate, the net NH_4_^+^ flux rate in the midrib, lateral vein, and shoot tip was increased by LT treatment (Figs. 1 and 2). This result was inconsistent with the uptake and distribution of ^15^N-NH_4_^+^ in the leaf and shoot tip (Table 1, Figs. 3 and 4). We selected the petiole and midrib as target tissues to study the effect of low temperature on the relative expression of nitrate and ammonium transporter genes.

Nitrate uptake by plants is regulated by transcriptional regulation [40]. Two environmental factors, temperature and nutrient concentration, significantly influence the expression of nutrient transporter genes [41]. However, few studies have examined the function of cucumber N transporters to date [42–44]. Little information on the regulatory pathways involved in the effect of low temperature on the expression of N transporter genes in cucumber has been reported. Among nitrate transporters, NRT1.1 (NPF6.3) is regarded to be a dual-affinity nitrate transporter that participates in nitrate absorption and transport [45, 46]. The present results showed that *CsNRT1.1* may not be the dominant gene involved in nitrate transportation in the petiole and midrib of cucumber. In Arabidopsis *AtNRT1.8* is associated with stress-induced NO_3_^−^ redistribution [47]. Under LT treatment, the relative expression of *CsNRT1.8* was up-regulated in the petiole and midrib. This response may allow an increase in nitrate transportation to the root, thus reducing the net ion flux rate in the petiole and midrib. In Arabidopsis *AtNRT1.4* and *AtNRT1.7* are responsible for nitrate transport to the petiole and leaf [48, 49]. We identified two homologs of *AtNRT1.4* in cucumber, *CsNRT1.4a* and *CsNRT1.4b*. Compared with that under NT treatment, the expression of *CsNRT1.4a* in the petiole and midrib under LT treatment was down-regulated, whereas the expression of *CsNRT1.4b* was up-regulated. The different responses in relative expression level of these genes to low temperature may reflect their different functions. *CsNRT1.7* is involved in NO_3_^−^ recycling in cucumber [42]. Under low temperature *CsNRT1.7* in the petiole and midrib was up-regulated. This response may reduce NO_3_^−^ upward transport to the leaves to some extent. *AtClCa* and *AtCLCe* are critical for nitrate transport into vacuoles in Arabidopsis [50, 51]. In the present experiment, the relative expression levels of *CsCLCa* and *CsCLCe* in the petiole and midrib of cucumber seedlings were up-regulated by low temperature. This response may lead to increased nitrate storage in vacuoles under low temperature. The AMT1 subfamily of Arabidopsis plays an important role in the stage of NH_4_^+^ absorption [52]. The MEP subfamily (*AtAMT2*) may play a role in the transport of NH_4_^+^ from the apoplast to the symplast [53]. In the present experiment, up-regulation of *CsAMT1.2a*–*1.2c* in the midrib may have contributed to the higher net NH_4_^+^ flux rate under low temperature.

### Under low temperature a higher proportion of NO_3_^−^ was reduced to NH_4_^+^ during its transportation in the stem and petiole

Under low temperature, although the total amount of NO_3_^−^-N and NH_4_^+^-N absorbed by cucumber seedlings and transported from the root to the shoot decreased, the net NH_4_^+^ flux rate increased in the midrib, lateral vein, and shoot tip. To elucidate the mechanism responsible, the morphological changes in N during the transport process were studied.

Nitrate reductase is the key rate-limiting enzyme in nitrate reduction [10]. In higher plants the activity of NR is regulated at the phosphorylation and transcriptional levels [54]. Under low temperature, NRA_max_ in the root decreased, whereas NRA_act_ did not change significantly, which indicated that low temperature reduced the amount of enzyme protein but had no significant effect on the apparent activity of the enzyme (Fig. 6). Therefore, the amount of NR protein may be redundant in the root. The NRA_act_ and NRA_max_ in the stem and petiole increased significantly, which indicated that the change in enzyme protein content was consistent with the change in enzyme apparent activity, and NRA_act_ in the stem and petiole was predominantly regulated by low temperature at the transcriptional level. Compared with those under NT treatment, NRA_max_ in the midrib and blade under low temperature did not decrease, whereas NRA_act_ decreased significantly in the midrib and blade. This response indicated that the effect of low temperature on NRA_act_ in the midrib and blade may be predominantly through protein phosphorylation. Overall, low temperature had no effect on NRA_act_ in the root, but significantly increased NRA_act_ in the stem and petiole of cucumber seedlings. These changes may account for the increased proportion of NO_3_^−^ reduced to NH_4_^+^ during its transportation in the stem and petiole.

The qRT-PCR analysis of NR gene expression and NRA_max_ analysis indicated that *CsNR1* and *CsNR3* may be the dominant NR gene in the root and leaf, respectively, of cucumber seedlings (Figs. 6A, 7A, C).

### Biological significance of the increase in net NH_4_^+^ fluxes in vigorously growing tissues under low temperature

Plants transfer nutrients to young tissues and seeds under unsuitable environmental conditions [55]. This process has been an important adaptive strategy during terrestrial plant evolution. In the present experiment, although the uptake and upward transportation of NH_4_^+^ decreased under low temperature, the net NH_4_^+^ flux rate in the midrib, lateral vein, and shoot tip increased significantly. This response may be due to the transformation of NO_3_^−^ during transportation. A greater proportion of NO_3_^−^ was reduced to NH_4_^+^ during the upward transportation of NO_3_^−^ under low temperature. The energy consumption during the N transportation process was reduced accordingly. This strategy may be an adaptation of plant N transport to the decrease in energy supply under low-temperature stress. According to Han et al. [56], under low-temperature stress, the NO_3_^−^-N content and NR activities in tomato leaves significantly decrease, whereas the NH_4_^+^-N content significantly increases. Under drought stress, NH_4_^+^ nutrition can limit the effect of water deficit by osmotic adjustment and thereby limit oxidative damage [57]. Therefore, assuming that NH_4_^+^ plays a role in the prevention of stress-induced peroxidation, the increase in NH_4_^+^ content in leaves and young tissues is not only beneficial for utilization of N nutrition, but also improves the stress tolerance of the plant.

The predominant N sources of cucumber are NO_3_^−^-N and NH_4_^+^-N. Compared with a single NO_3_^−^-N source or a single NH_4_^+^-N source, a compound N source is more conducive to N absorption and plant growth [31]. Plant preference for NO_3_^−^-N or NH_4_^+^-N is associated with species and is influenced by environmental conditions and growth stage [58–60]. Kant [61] considered that improvement of nitrate uptake and transport would enhance plant growth, resulting in improved crop yields. In the future, further in-depth research on nitrate and ammonium transport in the roots of cucumber seedlings and research on enhancing the N nutrition status of plants by improving the ratio of NO_3_^−^-N to NH_4_^+^-N under low temperature is required.

## Conclusions

**Fig. 8:**
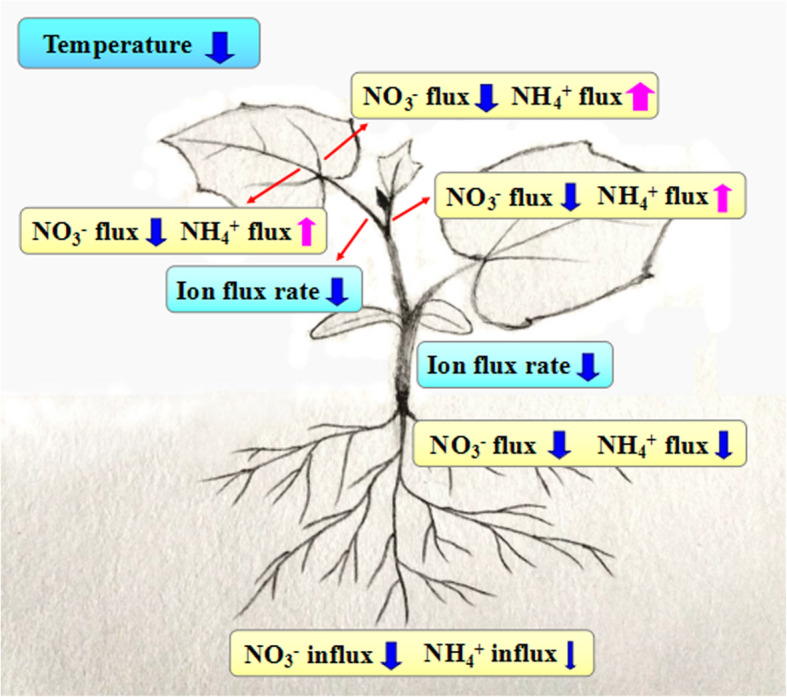
Regulation of low temperature on net NO_3_^−^ and NH_4_^+^ flux rates in cucumber seedlings. The color of the arrows indicates the changes in relevant indicators: pink indicates increase, and blue indicates decrease. The thickness of the arrows indicates the degree of increase or decrease

Our results provide evidence that cucumber seedlings reduce a greater proportion of NO_3_^−^ to NH_4_^+^ during the process of upward transport of NO_3_^−^ under low temperature. This action may reduce the dependence of N transport on energy and enable plants to adapt to the decrease in energy supply under low-temperature stress. Compared with NO_3_^−^, the absorption of NH_4_^+^-N and net NH_4_^+^ flux rate in the root hair zone, vascular bundles of the primary root, and the stem is less inhibited by low temperature (Fig. 8). Under low temperature, the net NH_4_^+^ flux rate in vascular bundles of the midrib, lateral vein, and shoot tip is increased, which is the opposite response to that of net NO_3_^−^ flux rate. In line with these responses, under low temperature, the relative expression of *CsNRT1.4a* in the petiole and midrib is down-regulated, whereas the expression of *CsAMT1.2a*–*1.2c* in the midrib is up-regulated. The NRA_act_ in the stem and petiole increases significantly, which is predominantly regulated at the transcriptional level by low temperature. Given the importance of cucumber as a greenhouse vegetable crop, this study enhances understanding of the low-temperature tolerance of a thermophilic plant and contributes to improved winter cultivation techniques of tender vegetables in a greenhouse.

## Methods

### Plant materials and growth conditions

All experiments in this study were conducted in controlled-environment chambers (Memmert ICH L260). Seeds of cucumber (*Cucumis sativus* L.) ‘Xintai Mici’ (China Vegetable Seed Technology Co., Ltd., Beijing, China) were incubated in the dark until germination at 28 °C. The seedlings were grown in a vermiculite–sand mixture (1:2, v/v) and supplied with half-strength modified Hoagland’s nutrient solution at 26 °C/17 °C (day/night) [26]. The photosynthetic photon flux density, photoperiod, and relative humidity (RH) were 350 μmol·m^− 2^·s^− 1^, 12 h/12 h (light/dark), and 70–80%, respectively. When the cotyledons of the seedlings were fully expanded, the seedlings were supplied with full-strength modified Hoagland’s nutrient solution (pH 6.0) containing 4 mM Ca (NO_3_)_2_, 5 mM KNO_3_, 1 mM NH_4_NO_3_, 1 mM KH_2_PO_4_, 2 mM MgSO_4_·7H_2_O, 40 μM EDTA-Fe, 4 μM H_3_BO_3_, 2 μM MnSO_4_·4H_2_O, 2 μM ZnSO_4_·7H_2_O, 1 μM CuSO_4_·5H_2_O, and 0.5 μM (NH_4_)_6_Mo_7_O_24_·4H_2_O. When the second leaves were fully expanded, the seedlings were used for the following experiments. In all experiments performed in this study, low temperature was set to 8 °C in accordance with Lee et al. [62].

#### Experiment 1: effect of low temperature on the net fluxes of NO_3_^−^ and NH_4_^+^, activities of NR and NiR, and gene expression in cucumber seedlings

The seedlings were divided into two groups and exposed to either normal temperature (NT; 26 °C) or low temperature (LT; 8 °C) for 5 h. During treatment, the light intensity and RH were identical to the seedling growth conditions. After treatment, the seedlings were harvested for physiological and genetic analyses.

#### Experiment 2: effect of low temperature on the uptake of NO_3_^−^-N and NH_4_^+^-N in cucumber seedlings

Uptake of NO_3_^−^-N and NH_4_^+^-N was measured in accordance with the method described by Garnett et al. [63] with some modifications. Briefly, the seedlings were transplanted into rectangular hydroponic containers and supplied with full-strength modified Hoagland’s nutrient solution 1 d prior to analysis. The containers were supplied with air bubblers to ensure adequate oxygen supply. On sampling days, plants were transferred to the same solution supplemented with ^15^N-labeled NO_3_^−^ or NH_4_^+^. The treatments were as follows:

1. NT: 26 °C ^15^N-labeled NO_3_^−^ (^15^N 25%)
2. LT: 8 °C ^15^N-labeled NO_3_^−^ (^15^N 25%)
3. NT: 26 °C ^15^N-labeled NH_4_^+^ (^15^N 100%)
4. LT: 8 °C ^15^N-labeled NH_4_^+^ (^15^N 100%)

After exposure for 5 h, the seedlings were harvested for the determination of ^15^N-NO_3_^−^ and ^5^N-NH_4_^+^ contents.

### Measurement of net NO_3_^−^ and NH_4_^+^ fluxes

**Fig. 9:**
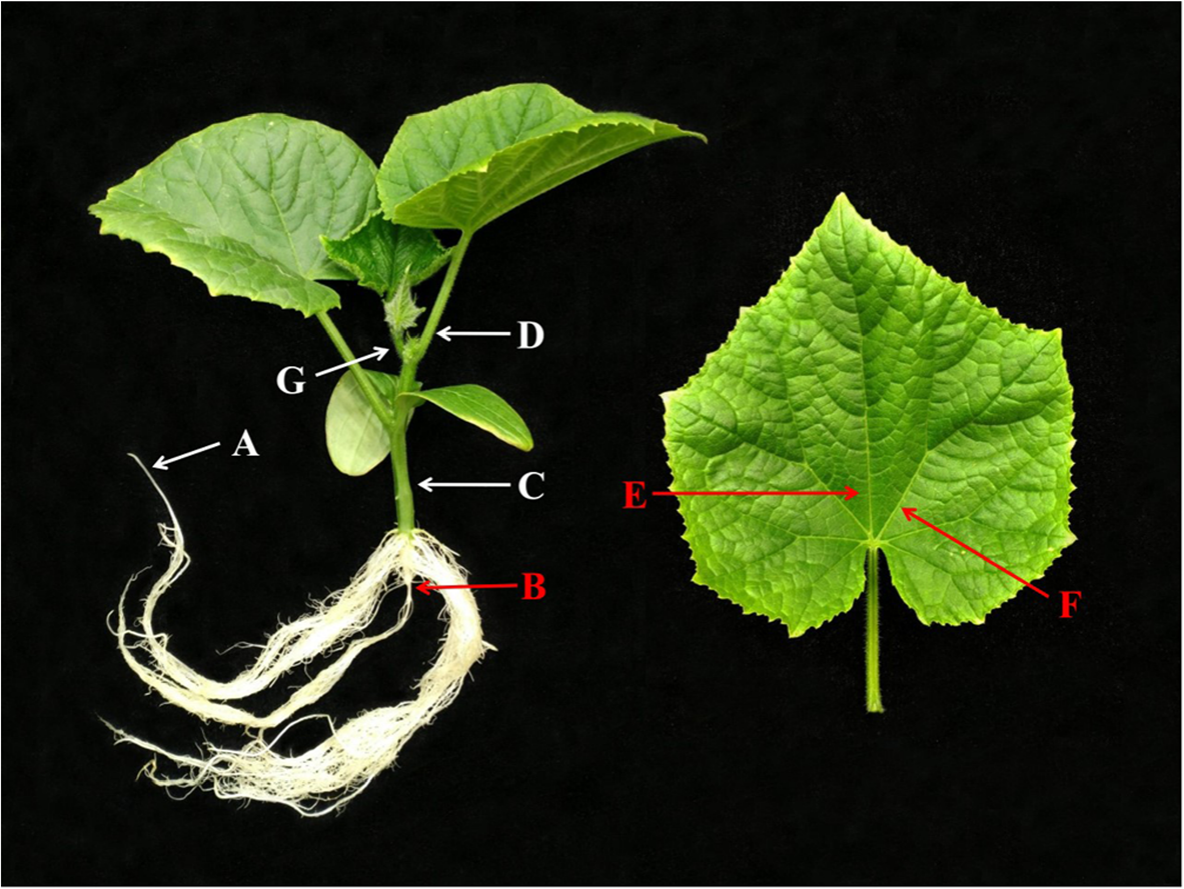
Location of samples from cucumber seedlings used for NMT measurement. **a** Root hair zone (the measurement site was 1500 μm from the first root hairs at the root apex, selected based on Additional file 3), **b** primary root, **c** stem, **d** petiole, **e** midrib, **f** lateral vein, **g** shoot tip

The net NO_3_^−^ and NH_4_^+^ fluxes were measured at the YoungerUSA Xuyue (Beijing) BioFunction Institute using a NMT system (NMT100 Series, YoungerUSA, LLC, Amherst, MA, USA; Xuyue (Beijing) Science & Technology Co., Ltd., Beijing, China) and imFluxes V2.0 software (YoungerUSA, LLC). The method followed that of Lei et al. [64] with some modifications. The root sample was excised about 2 cm from the apex. The primary root, stem, petiole, midrib, lateral vein, and shoot tip were sampled at the positions indicated in Fig. 9. A seedling was only used once. The sample was fixed with a belt. The transverse section of each sampled organ was immediately incubated in the measuring solution (1.625 mM Ca (NO_3_)_2_, 0.25 mM NH_4_NO_3_, 0.1 mM MgSO_4_, and 0.3 mM MES; pH 6.0) to equilibrate for 15 min because a rapid and large efflux of NO_3_^−^ and NH_4_^+^ occurred after the samples were excised. The flux rate gradually decreased and stabilized within 15 min. The solution was then sucked out and fresh measuring solution was injected. Uptake of NO_3_^−^ and NH_4_^+^ was measured in the root hair zone (Figs. S1, S2). Transport of NO_3_^−^ and NH_4_^+^ was measured in the transverse sections of the organs. The microsensor was fixed at the center of the vascular bundle (Figs. S2, S3). The excised sections were incubated in the measuring solution throughout the experiment.

A pre-pulled and silanized microsensor (Φ4.5 ± 0.5 μm, XY-CGQ-01, YoungerUSA) was first filled with a backfilling solution (NO_3_^−^: 10 mM KNO_3_; NH_4_^+^: 100 mM NH_4_Cl) to a length of approximately 1.0 cm from the tip. The micropipettes were front-filled with 50–80 μm columns of selective liquid ion-exchange cocktails (NO_3_^−^: NO_3_^−^ LIX, XY-SJ-NO_3_; NH_4_^+^: LIX, XY-SJ-NH_4_^+^; YoungerUSA). An Ag/AgCl wire microsensor holder (YG003-Y11, YoungerUSA) was inserted in the back of the microsensor to make electrical contact with the electrolyte solution. The microsensor holder was used as the reference microsensor. The microsensor was calibrated before and after flux measurements with culture media that differed in concentrations of NO_3_^−^ and NH_4_^+^ (NO_3_^−^: 5.0 mM and 0.5 mM; NH_4_^+^: 0.5 mM and 0.1 mM). Only a microsensor with an absolute value of the Nernstian slope > 50 mV decade^− 1^ was used. Data were discarded if the post-test calibrations failed. The net NO_3_^−^ and NH_4_^+^ fluxes were calculated using JCal V3.3 software (a free MS Excel spreadsheet; YoungerUSA, LLC).

### Measurement of NO_3_^−^-N and NH_4_^+^-N uptake

After treatment for 5 h, roots of the seedlings in Experiment 2 were rinsed for 2 min in identical, unlabeled modified Hoagland’s nutrient solution. The root surface was dried with absorbent paper. In addition, the root, stem, cotyledon, first petiole, first blade, second petiole, second blade, and shoot tip were sampled, respectively. All samples were defoliated at 105 °C and dried to constant weight at 55 °C. The fresh weight, dry weight, and water content of the samples were determined in accordance with Oliviero et al. [65]. The samples were ground to a fine powder. Total N and ^15^N contents were measured by means of continuous-flow isotope-ratio mass spectroscopy using a vario PYRO cube elemental analyzer coupled to an IsoPrime 100 isotope-ratio mass spectrometer [66]. During the analysis process, 12 samples were interspersed with a laboratory sample for correction.

### Detection of NR and NiR activities

#### Measurement of NR activity

The NR activity was measured using the method described by Reda and Klobus [67] with some modifications. Plant tissues (1.0 g fresh weight) were ground in a chilled mortar with 5 mL extraction buffer. The mixture was homogenized and centrifuged at 15000 *g* for 10 min at 4 °C. The supernatant was used to measure NR activity in the presence and/or absence of MgCl_2_ in accordance with the method of Glaab and Kaiser [68]. The reaction medium was incubated for 10 min at 27 °C, and then the NR activity was recorded by measuring the NO_2_^−^ produced.

#### Measurement of NiR activity

The NiR activity was measured following the method described by Liu et al. [69].

### qRT-PCR analysis

The samples were excised, frozen in liquid nitrogen and stored at − 80 °C for quantification of the expression of nitrate transporter family genes (*CsNRT1.1*, *CsNRT1.2a*–*CsNRT1.2c*, *CsNRT1.3*, *CsNRT1.4a*–*CsNRT1.4b*, *CsNRT1.5a*–*CsNRT1.5c*, and *CsNRT1.7*–*CsNRT1.10*), chloride channel protein family genes (*CsCLCa*–*CsCLCg*), slow anion channel-associated homologs (*CsSLAH1*–*CsSLAH4*), ammonium transporter family genes (*CsAMT1.2a*–*CsAMT1.2c*, *CsAMT2*, and *CsAMT3.3*), NR family genes (*CsNR1*–*CsNR3*), and a NiR gene (*CsNiR*).

Total RNA was extracted using the RNAprep pure Plant Kit (TIANGEN, Beijing, China) in accordance with the manufacturer’s instructions. The concentration of RNA was quantified by spectrophotometric measurement at λ = 260 nm and RNA integrity was checked on agarose gels [70]. First-strand cDNA was synthesized using the FastQuant RT Kit (TIANGEN) following the manufacturer’s instructions. The cDNA was analyzed by qRT-PCR using the Hieff qPCR SYBR Green Master Mix (11203ES03, YEASEN) on an ABI 7500 Real Time PCR System (Applied BioSystems) [71]. Transcripts of *TIP41* (*PP2A phosphatase activator*; GW881871) were used to standardize the cDNA samples for different genes because its expression is insensitive to low temperature [72]. Specific primers were designed using Primer Premier 5 software [73] and the cucumber genome database [1]. Oligonucleotides used are listed in Additional file 1 (Table S1).

### Data analysis

Two-way analysis of variance (ANOVA) followed by the least significant difference test was performed. All statistically significant differences were identified as *P* < 0.05. Graphpad Prism 5 was used for graphical presentation.

## Supplementary Information

**Additional file 1: Table S1.** Oligonucleotides used in the study.

**Additional file 2: Table S2.** Effect of low temperature on the water content of cucumber seedlings.

**Additional file 3: Fig. S1.** Net NO_3_^−^ and NH_4_^+^ flux rates at different positions in the root hair zone of cucumber seedlings.

**Additional file 4: Fig. S2**. Positions of electrode pole against tissues during the test.

**Additional file 5: Fig. S3.** Vascular bundles in the primary root, stem, petiole, midrib, and lateral vein for the NMT test.

## Data Availability

The datasets used and analyzed during the current study are available from the corresponding author on reasonable request.

## Abbreviations

NT: Normal temperature, 26 °C
LT: Low temperature, 8 °C
NMT: Non-invasive micro-test technology
NR: Nitrate reductase
NiR: Nitrite reductase
NRT: Nitrate transporter
AMT: Ammonium transporter
MEP: Methylammonium permease
NRA: Nitrate reductase activity
NRA_act_: Actual nitrate reductase activity
NRA_max_: Maximum nitrate reductase activity
NRT1: Nitrate transporter 1 family
NRT2: Nitrate transporter 2 family
CLC: Chloride channel family
SLAH: Slow anion channel-associated homologs
