# Supplementary material for: Adaptation of cucumber seedlings to low temperature stress by reducing nitrate to ammonium during it’s transportation
Source: BMC Plant Biol. 2021 Apr 19;21:189. doi: 10.1186/s12870-021-02918-6 (PMC8056598; doi:10.1186/s12870-021-02918-6)
Supplement: Supplementary file 1 — Additional file 1: Table S1. Oligonucleotides used in the study. [file 12870_2021_2918_MOESM1_ESM.docx]

**Table S1. Oligonucleotides list used in the study**

| **Cucumber gene** | **Gene number** | **Forward primer** (5’-3’) | **Reverse primer** |
| --- | --- | --- | --- |
| *CsTIP41* | CsaV3_7G008050 | CAACAGGTGATATTGGATTATGATTATAC | GCCAGCTCATCCTCATATAAG |
| *CsNRT1.1* | CsaV3_3G002970 | TGGTGTCGGTTTAGTTCTGTCG | TGTAGATAGCCCTGTGCTCATTGTT |
| *CsNRT1.2a* | CsaV3_5G032350 | TGAAGACAACAAAGGGTGGGA | TGAGTTTCGTGAGTTCGTGCC |
| *CsNRT1.2b* | CsaV3_1G002140 | AGATTCTCCTGACCCTTTGCC | CAGAAGCCTTAGTAACACCATTGAC |
| *CsNRT1.2c* | CsaV3_6G036160 | CGCTTCCCATTACGTTCCT | CTATGAGGTCTTTGGCTACGG |
| *CsNRT1.3* | CsaV3_2G034610 | AAGTATCGGGTCGTTGTTTGC | GGGCTTCCTCTTGGCTTTT |
| *CsNRT1.4a* | CsaV3_2G002800 | CGTTACCACAACTACATCCACCTC | GCCCAAATCCCGAAACACT |
| *CsNRT1.4b* | CsaV3_1G030010 | GGGAGCCCTGTAGTTCATATTT | CGTGAAGCTTGATCAGGAGAA |
| *CsNRT1.5a* | CsaV3_3G010210 | GCCCTATGTATGACCTCGATTT | ATCCATCCCGGCATGTTATC |
| *CsNRT1.5b* | CsaV3_3G049430 | GGTCAATGAACCGTGGTAGAAG | TGGTGGAGAGCCAAATAGGAA |
| *CsNRT1.5c* | CsaV3_1G042330 | CTCAATCACCTGACGGAATC | CTCCACATGACCCGAATTTA |
| *CsNRT1.7* | CsaV3_1G002110 | TAACTCCAAACTTTCCTATTCA | CCAAACTGGTAGCACTCTCACT |
| *CsNRT1.8* | CsaV3_3G001980 | ACCTTTCCATTTACCTCACAGC | ATCATCAAACAACCCTTCTCCT |
| *CsNRT1.9* | CsaV3_1G002120 | GCGGCATTACGCTCCTACA | TGAACGCCTCTGACAACCC |
| *CsNRT1.10* | CsaV3_3G049290 | TGTCCAGCAAAGAGCGGTAA | AATCGGGTGGCACAAAGC |
| *CsCLCa* | CsaV3_2G034160 | CAATGATCTCGCTACCCTTCTT | GACAAGGGAAAGAGGCTGATAC |
| *CsCLCc* | CsaV3_3G006610 | TATTGGCGGTGTGTTTGG | CGGCAACGACAAGGATAAG |
| *CsCLCd* | CsaV3_6G038240 | CTTCCATATCTGAACCCATCTC | CATCTGAATCTTCGCTATCCTC |
| *CsCLCe* | CsaV3_6G009920 | GCATTCAAGGTCCCAGTTTA | TCTCGGTGCACCAAATTC |
| *CsCLCf* | CsaV3_3G003830 | GTGTGTGGAGGTGTTATAGTG | CGAACAGCCAGTACCTAAAG |
| *CsCLCg* | CsaV3_2G003210 | GCTGTAGTTGCTGTGGTATT | TCCAAGTATGCCTCCGATAA |
| *CsSLAH1* | CsaV3_7G003500 | CTAATCCCAGTAGCCAACTTTC | GGAGCTGCAAAGAACAAGA |
| *CsSLAH2* | CsaV3_5G000940 | CTTCTTTGCACCCTGGATAG | TTTCGACAGCCTTCGTTG |
| *CsSLAH3* | CsaV3_3G003380 | CTGTTTGGTGTGCCTTCAT | CCTCCAACCATCCACATTTC |
| *CsSLAH4* | CsaV3_3G045580 | GGCCGGTGTTCTTTCTTT | TTACCAGGGCGGTGAATA |
| *CsNRT2.1* | CsaV3_1G004650 | GCCCCATTTCAACAATACCCT | TCCTGGAGAACCTTCAACATCAC |
| *CsNRT2.2* | CsaV3_1G013370 | GCGTCTGTTTCTGGGAGTATCTT | CCGCAAGCCCTATTATCTGACTA |
| *CsNRT2.3* | CsaV3_5G031350 | ATCGTCTCCCGAAGGTCACTA | GCAGGCTATTATCATCACTCCC |
| *CsAMT1.1a* | CsaV3_2G012160 | GCCGATTACAGCTTCTTTCT | AGAACCAATGGGACACAAC |
| *CsAMT1.1b* | CsaV3_3G011530 | CCCTTTCAATCCAGGTTCGT | GTGGTGGTTACTGCTGTTCGTC |
| *CsAMT1.2a* | CsaV3_3G028490 | CTCCTCTGGTTCGGCTGGTA | AGTGGTAAATGCGGCTGTGC |
| *CsAMT1.2b* | CsaV3_2G010060 | ATCGCCCTTACTACGGTCAAT | TACAACGGCACAACCAGACG |
| *CsAMT1.2c* | CsaV3_2G010040 | GTCGACAACAACTACCTTCTC | CAAAGGCGAAACCGAAGA |
| *CsAMT2* | CsaV3_3G033490 | AGAGTGGCGTGAACGATGGA | GCCGACGGTGTATGAGAAAA |
| *CsAMT3.3* | CsaV3_6G047560 | GCCTGATGTATCCAGATTCCCTAT | GATAAACACTGCTCCTGCGAACT |
| *CsNR1* | CsaV3_4G028560 | TCCCACCAATCATCTCCTAATC | CAATCAGCCGTTCCGAAATC |
| *CsNR2* | CsaV3_5G023370 | TACTGGTGCTGGTGTTTCTGGT | GATTCCGATTTCTCCCTTGTGA |
| *CsNR3* | CsaV3_5G023350 | TCATCACCCAAAGCAGTTACTC | CTTTCCAACGGGTAGTCCTAATC |
| *CsNiR* | CsaV3_3G018610 | CATTTCTGTCTCCTCCTGTAGC | CACCACCCAATATCCATCTCTC |
