# Supplementary material for: Adaptation of cucumber seedlings to low temperature stress by reducing nitrate to ammonium during it’s transportation
Source: BMC Plant Biol. 2021 Apr 19;21:189. doi: 10.1186/s12870-021-02918-6 (PMC8056598; doi:10.1186/s12870-021-02918-6)
Supplement: Supplementary file 2 — Additional file 2: Table S2. Effect of low temperature on the water content of cucumber seedlings. [file 12870_2021_2918_MOESM2_ESM.docx]

**Table S2. Effect of low temperature on the water content in cucumber seedlings**

| Water content % | Root | Stem | Cotyledon | First petiole | First blade | Second petiole | Second blade | Shoot tip |
| --- | --- | --- | --- | --- | --- | --- | --- | --- |
| NT (26℃) | 95.60±0.15 a | 95.41±0.13 a | 89.33±0.19 a | 95.41±0.13 a | 86.83±0.16 a | 95.16±0.38 a | 87.18±0.12 a | 88.24±0.14 a |
| LT (8 ℃) | 95.61±0.25 a | 95.43±0.15 a | 89.52±0.13 a | 95.20±0.11 a | 86.63±0.17 a | 95.29±0.24 a | 86.96±0.16 a | 88.22±0.10 a |

Values were means ± SE (n = 3). Different lower-case letters indicate a significant difference (*P <* 0.05).
