# Supplementary material for: Adaptation of cucumber seedlings to low temperature stress by reducing nitrate to ammonium during it’s transportation
Source: BMC Plant Biol. 2021 Apr 19;21:189. doi: 10.1186/s12870-021-02918-6 (PMC8056598; doi:10.1186/s12870-021-02918-6)
Supplement: Supplementary file 3 — Additional file 3: Fig. S1. Net NO3− and NH4+ flux rates at different positions in the root hair zone of cucumber seedlings. [file 12870_2021_2918_MOESM3_ESM.docx]

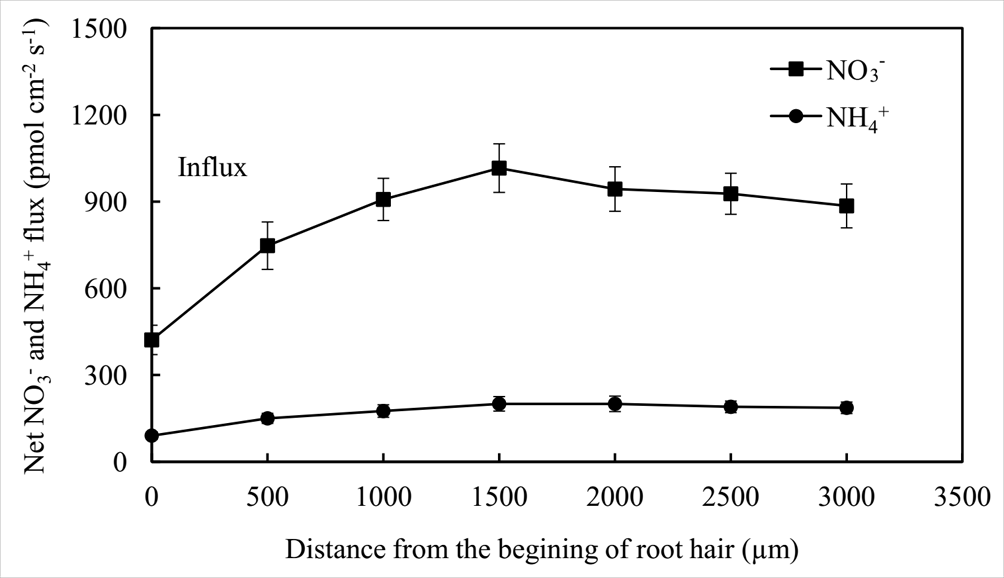


**Fig. S1.** Net NO_3_^-^ and NH_4_^+^ flux rate at different positions in root hair zones of cucumber seedling. NO_3_^-^ and NH_4_^+^ fluxes were measured along the root axes (0–2500 μm from the first root hairs) at intervals of 500 μm. Based on this, the point at 1500 μm from the first root hairs was selected as the detection location. Error bars represent the standard error of the mean (n = 5).
