# Supplementary material for: Adaptation of cucumber seedlings to low temperature stress by reducing nitrate to ammonium during it’s transportation
Source: BMC Plant Biol. 2021 Apr 19;21:189. doi: 10.1186/s12870-021-02918-6 (PMC8056598; doi:10.1186/s12870-021-02918-6)
Supplement: Supplementary file 4 — Additional file 4: Fig. S2. Positions of electrode pole against tissues during the test. [file 12870_2021_2918_MOESM4_ESM.docx]

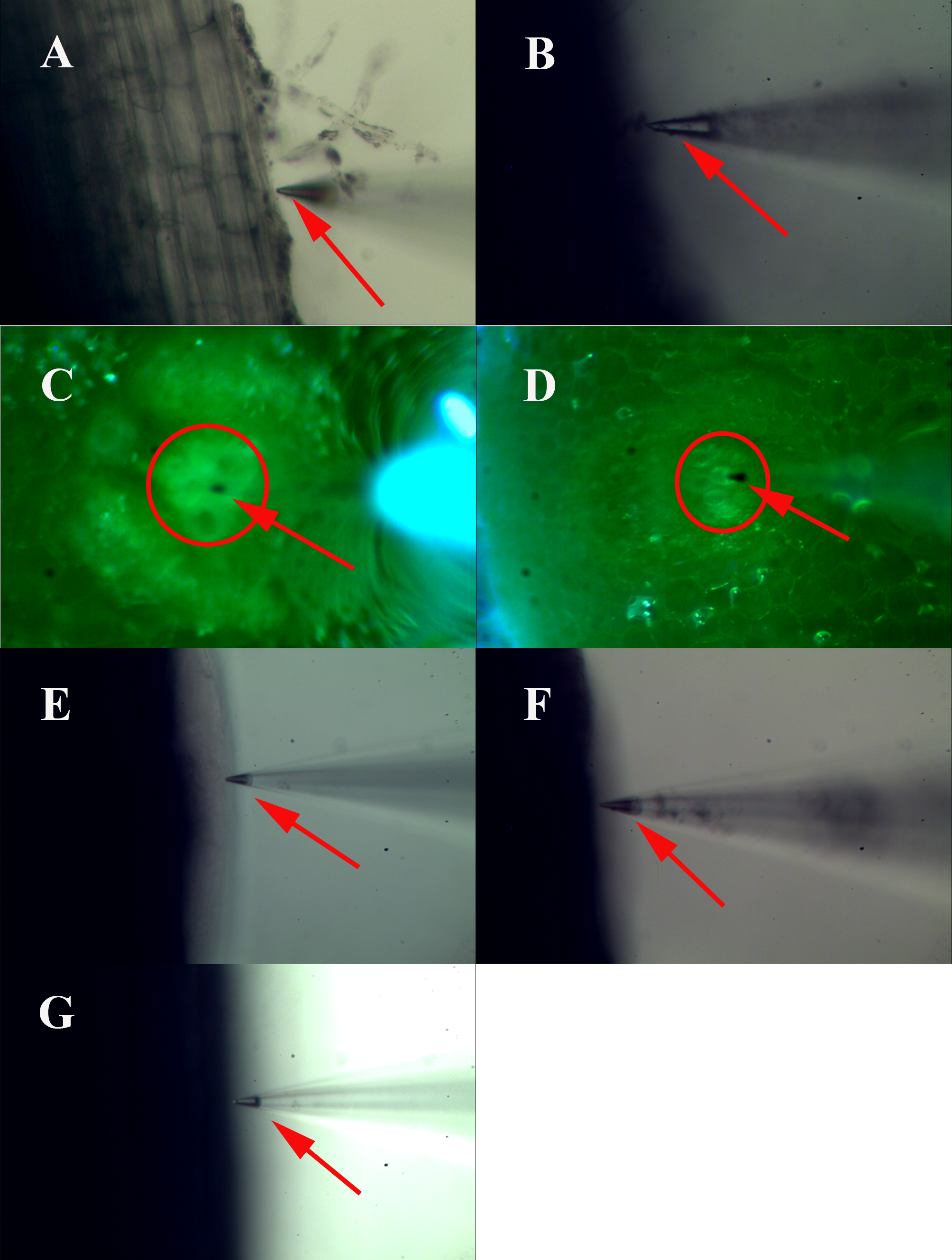


**Fig. S2.** Positions of electrode pole against tissues during the test.

(A) root hair zone. The measuring sight was 1500 μm from the first root hairs, selected based on Figure S1. When detecting the net NO_3_^-^ and NH_4_^+^ fluxes rate in B-F, the electrode pole was aligned with the center of vascular bundle cross section. (B) primary root. (C) stem. (D) petiole. (E) midrib. (F) lateral vein. (G) shoot tip: In this test, the electrode was aligned with the center of the shoot tip cross section. Inverted microscope was used to detect A, B, E, F, and G. Stereo microscope was used to detect C and D. Where the red arrow points is the electrode. In the red circles are the vascular bundles of the stem (C) and petiole (D) respectively.
