# Supplementary figures and images for: Adaptation of cucumber seedlings to low temperature stress by reducing nitrate to ammonium during it’s transportation
Source: BMC Plant Biol. 2021 Apr 19;21:189. doi: 10.1186/s12870-021-02918-6 (PMC8056598; doi:10.1186/s12870-021-02918-6)

**
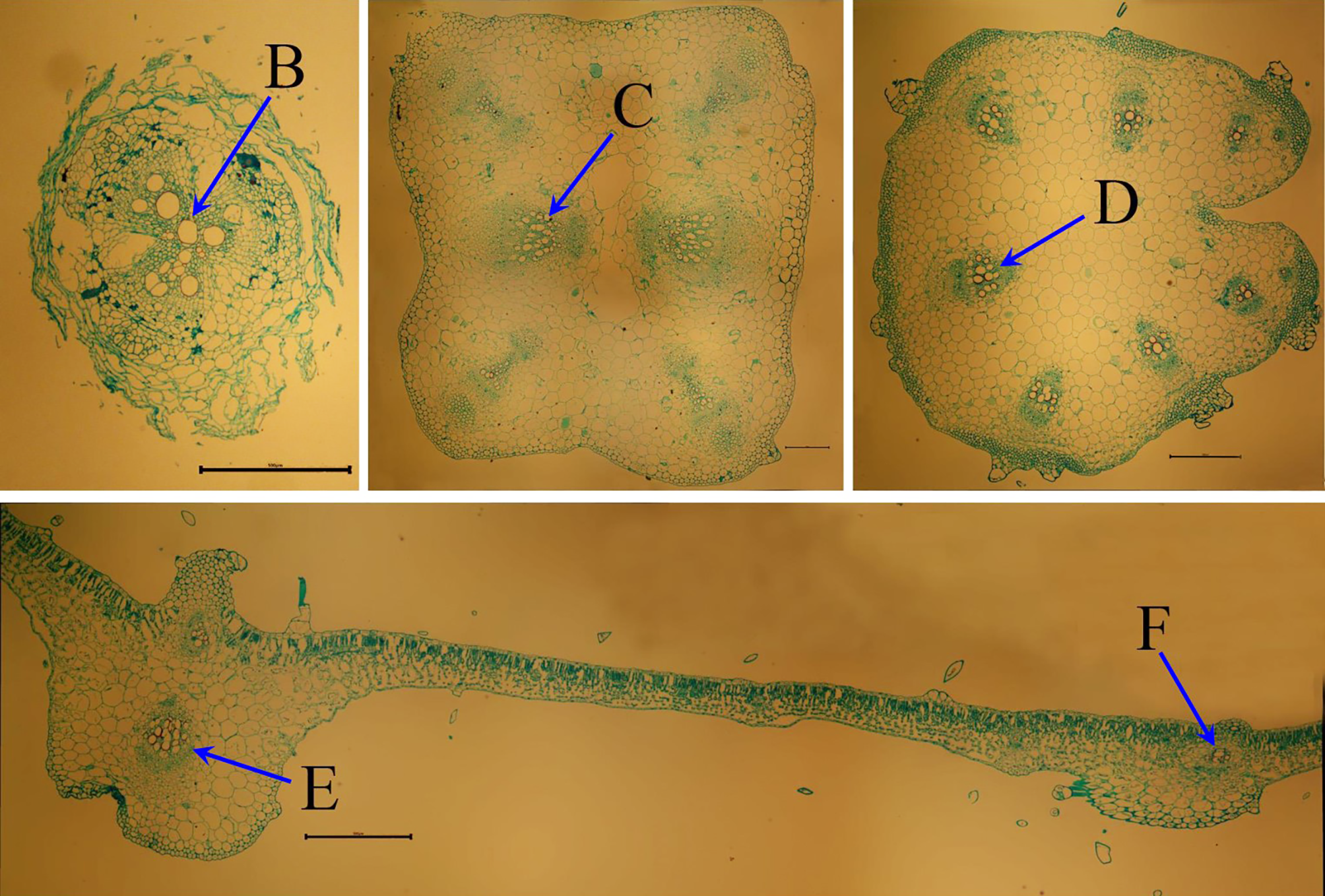
**

**Fig. S3.** Vascular bundles in primary root (B), stem (C), petiole (D), midrib (E), and lateral vein (F) for NMT test.

Supplement: Supplementary file 5 — Additional file 5: Fig. S3. Vascular bundles in the primary root, stem, petiole, midrib, and lateral vein for the NMT test. [file 12870_2021_2918_MOESM5_ESM.docx]
